# Role of the Bishop Score in Predicting Successful Induction of Vaginal Delivery: A Systematic Review of Current Evidence

**DOI:** 10.7759/cureus.87467

**Published:** 2025-07-07

**Authors:** Antonios Michail, Zacharias Fasoulakis, Ekaterini Domali, George Daskalakis, Panagiotis Antsaklis

**Affiliations:** 1 1st Department of Obstetrics and Gynecology, General Hospital of Athens ‘Alexandra Hospital’, National and Kapodistrian University of Athens, Athens, GRC; 2 Obstetrics and Gynecology, Democritus University of Thrace, Alexandroupolis, GRC

**Keywords:** bishop score, cervical ripening, labor induction, predictive models, vaginal delivery

## Abstract

Labor induction is a common obstetric intervention, and the Bishop score remains a widely used clinical tool for predicting its success by assessing cervical readiness. This systematic review synthesizes evidence from 36 studies published between 2013 and 2025 across diverse populations to evaluate the predictive value of the Bishop score for successful vaginal delivery following labor induction. The analysis highlights that a Bishop score below 6 typically necessitates cervical ripening with pharmacologic and mechanical methods demonstrating variable efficacy based on initial cervical conditions. Prostaglandins, particularly dinoprostone and misoprostol, show superior effectiveness in women with unfavorable scores (≤3-4), while simpler interventions suffice when scores are ≥6. The integration of additional predictors, including ultrasound-based parameters (cervical length, uterocervical angle) and biochemical markers (fetal fibronectin, IGFBP-1), has demonstrated improved prognostic accuracy. Machine learning models combining clinical and sonographic data further enhance prediction and guide individualized induction strategies. Despite the Bishop score’s continued clinical relevance, limitations in its predictive capacity, particularly in heterogeneous populations, highlight the need for refined, multiparametric tools. In the context of vaginal birth after cesarean (VBAC), Bishop score contributes to risk stratification and induction planning, but its predictive value must be contextualized within broader clinical factors. Overall, the Bishop score remains a valuable component of labor management; however, the evolving landscape of obstetric care calls for its integration with modern technologies and evidence-based adjuncts to optimize induction outcomes and ensure patient-centered decision-making.

## Introduction and background

Labor induction is one of the most frequently performed obstetric interventions and is undertaken when the benefits of delivery outweigh the risks of continued pregnancy [1]. The decision to induce labor is based on a range of maternal, fetal, and obstetric factors, and its success depends heavily on the condition of the cervix, the indication for induction, and the method employed [2, 3]. Successful induction not only reduces the need for cesarean delivery but also minimizes maternal and neonatal morbidity, whereas a failed induction is often associated with longer hospital stays, increased resource utilization, and adverse outcomes [4].

Cervical readiness plays a pivotal role in determining the likelihood of a successful induction of labor. A cervix that is favorable, meaning it is soft, anterior, dilated, and effaced with a well-applied presenting part, is more responsive to oxytocin or mechanical methods, reducing the risk of prolonged labor and cesarean delivery. To improve the prediction of induction outcomes, the Bishop score was introduced as a clinical tool to assess cervical readiness by incorporating five parameters: cervical dilatation, effacement, consistency, position, and fetal station [5]. While convenient and widely adopted, the Bishop score has recognized limitations, including interobserver variability and reduced predictive power in specific populations such as nulliparous women or those undergoing preterm induction [6]. Studies have suggested that additional factors, such as parity, gestational age, and maternal body mass index (BMI), may also play a crucial role in determining the likelihood of induction success, and new predictive models are being developed to address these complexities [7].

Globally, induction of labor rates have steadily increased, with estimates ranging from 20-30% in high-income countries, driven by both medical indications and elective choices. This growing clinical demand, alongside limitations of traditional assessment tools, has prompted the development of alternative or complementary methods, such as transvaginal ultrasound cervical length, elastography, and machine learning models, to improve prediction of induction outcomes. Recent advancements in ultrasound assessment, biochemical markers, and integrated scoring systems have prompted reevaluation of the Bishop score’s place in contemporary obstetric care [8, 9]. A comprehensive review of the latest literature is warranted to clarify the predictive value of the Bishop score in modern practice, to explore its potential limitations, and to determine whether alternative approaches offer greater clinical utility.

The aim of this review is to explore the role of the Bishop score in predicting the outcome of labor induction by synthesizing recent evidence from the literature. This includes evaluating its strengths and limitations, comparing it to emerging tools, and identifying innovations that may enhance predictive accuracy and support individualized obstetric care.

## Review

Materials and Methods

Study Design

This study is a structured literature review focused on examining the predictive value of the Bishop score in the context of labor induction. The review includes peer-reviewed studies that evaluate the use, reliability, and clinical outcomes associated with the Bishop score across various obstetric populations and induction methods. This systematic review was conducted in accordance with the Preferred Reporting Items for Systematic Reviews and Meta-Analyses (PRISMA) 2020 guidelines [10].

Search Strategy

A comprehensive literature search was conducted in the databases PubMed, Scopus, and Google Scholar to identify relevant articles published from January 2013 to May 2025. The search terms included combinations of: “Bishop score”, “labor induction”, “prediction of vaginal delivery”, “cervical assessment”, “induction success”, and “pre-induction scoring systems”. Boolean operators (AND, OR) were used to refine the search. Additional references were identified through backward citation tracking from key articles.

Inclusion and Exclusion Criteria

Studies were eligible for inclusion if they met the following criteria: a) Published in English; b) Included pregnant individuals undergoing labor induction; c) Assessed Bishop score as a predictive tool for induction success (vaginal delivery, cesarean rate, or induction-to-delivery interval) and d) Reported quantitative outcomes or comparative data.

Due to substantial clinical and methodological heterogeneity across studies, including variations in Bishop score thresholds, induction indications, gestational ages, and outcome definitions, we anticipated limitations in the ability to draw universal conclusions. This heterogeneity reflects underlying differences in the published literature rather than in study selection methodology.

Exclusion criteria included: (a) Editorials, commentaries, letters to the editor, and conference abstracts; (b) Studies that focused exclusively on non-obstetric populations or used the Bishop score outside the context of labor induction.

PRISMA process

**Figure 1:**
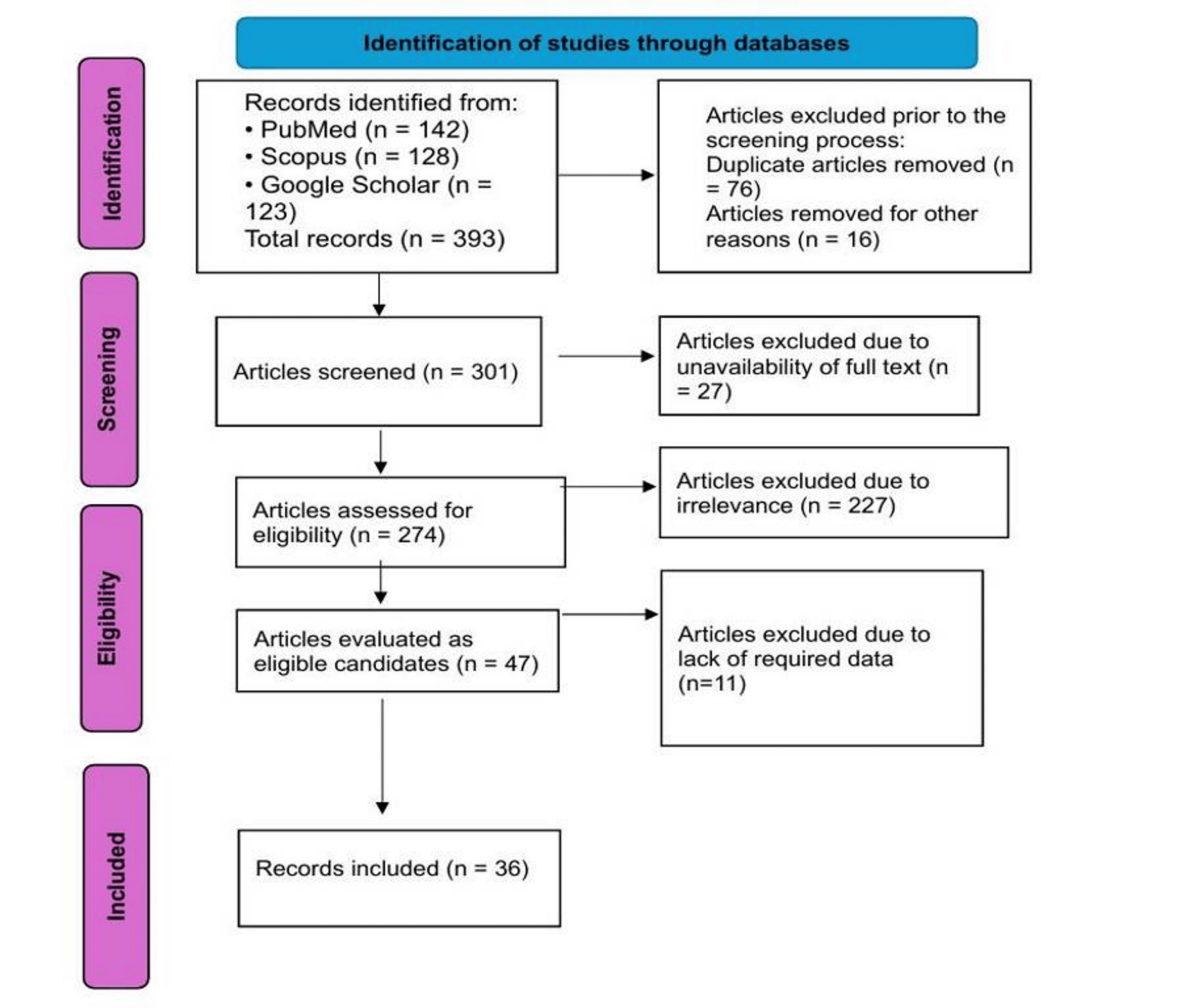
Flowchart of the study selection process.

A total of 393 articles were identified through database searches. Following the removal of 92 records, 301 articles remained for screening. During the screening phase, 227 records were excluded due to irrelevance to the research question, and an additional 27 articles were excluded due to unavailability of the full text. This left 36 full-text articles that were assessed for eligibility. No articles were excluded at this stage due to a lack of required data. Ultimately, 36 studies were included in the final analysis of the systematic review. The study selection process is illustrated in Figure 1.

Quality Assessment

The quality and risk of bias of the included studies were independently assessed by two reviewers using the Newcastle-Ottawa Scale (NOS) for observational studies [11]. Any disagreements were resolved through discussion or consultation with a third reviewer to ensure objectivity and consistency in the evaluation process.

Data Extraction and Analysis

Two reviewers independently extracted data from the eligible studies using a standardized extraction form. Extracted information included: authorship, year of publication, country of study, study design, population characteristics (e.g., parity, gestational age, maternal comorbidities), method of labor induction, Bishop score thresholds, reported outcomes such as vaginal and cesarean delivery rates, as well as predictive performance metrics (e.g., sensitivity, specificity, area under the curve) and NOS score.

Given the heterogeneity in study designs, induction protocols, and populations, a qualitative synthesis of findings was performed. Consistent patterns in the predictive value of the Bishop score and associated clinical outcomes were highlighted where appropriate. No formal meta-analysis was conducted due to variability in the data.

Results

This systematic review included studies from a wide range of countries, including the United States, South Korea, China, Malaysia, Turkey, Egypt, Brazil, Spain, Jordan, Israel, Serbia, India, Italy, France, Ethiopia, and Poland, spanning a publication period from 2013 to 2025. In total, 36 studies with varying sample sizes, ranging from 66 to 1408 participants, were analyzed to evaluate the role of Bishop score in predicting successful labor induction [12-47]. Several of these studies also examined additional predictive factors, such as cervical length, uterocervical angle (UCA), and other maternal or fetal parameters.

Across the 36 included studies, most inductions were performed at term gestation (≥37 weeks), with some studies including late preterm cases depending on clinical indication. Nearly all studies focused on singleton pregnancies, while multiple gestations were usually excluded. Nulliparous women were either the primary population or were analyzed separately to assess predictive accuracy by parity. Several studies excluded women with a prior cesarean, though others included VBAC cases as a specific subgroup. Indications for induction varied but commonly included post-term pregnancy, PROM, hypertensive disorders, and diabetes. Additional maternal characteristics, such as age and BMI, were reported inconsistently but were occasionally incorporated into multivariable analyses.

The studies included in the results synthesis are summarized in Table 1.

**Table 1:** Summary of the Included Studies. AOP: angle of progress; ART: artificial rupture of membranes; BMI: body mass index; CL: cervical length; fFN: fetal fibronectin; IOL: induction of labor; IUGR: intrauterine growth restriction; IV: intravenous; IU: international units; mIU: milli-international units; NOS: Newcastle-Ottawa Scale; NS: non-statistical; PCA: posterior cervical angle; PROM: premature rupture of membranes; psAOP: parasyphysis angle of progress; PGE1: prostaglandin E1; PGE2: prostaglandin E2; TPU: transperineal ultrasound; TVS: transvaginal sonography; TVUS: transvaginal ultrasound; UCA: uterocervical angle; VBAC: vaginal birth after cesarean

N	Study, Year	Country	Type of study	Number of Participants	Study Objective	Method of Induction of Labor	Results	NOS score
1	Zelig et al., 2013 [12]	USA	Retrospective cohort study	696	To determine the Bishop score that is most predictive of successful induction of labor (IOL) for different maternal weight categories.	N/A	The optimal Bishop score for predicting successful induction of labor (IOL) in nulliparous patients was 5 across all BMI categories. In the overall cohort (n = 696), patients with a Bishop score ≥5 had a significantly higher success rate compared to those with a score <5 (75% vs. 56%, χ² = 27.3, p < 0.0001). This threshold remained optimal within each BMI subgroup: among normal weight women, success rates were 79% versus 64% (χ² = 7.6, p = 0.006); among overweight women, 72% versus 58% (χ² = 4.8, p = 0.03); and among obese women, 73% versus 45% (χ² = 15.4, p < 0.0001)..	8/9
2	Yang et al., 2021 [13]	Korea	Prospective cohort study	205	To determine the value of the uterocervical angle (UCA) for predicting successful IOL in pregnant women, in comparison with the Bishop score and cervical length (CL).	Administration of dinoprostone for Bishop score < 4 and intravenous oxytocin; if Bishop score > 4, oxytocin only.	Compared to the unsuccessful IOL group, women in the successful IOL group had a significantly wider UCA (p = 0.012) and higher Bishop score (p = 0.001). However, CL was not significantly different (p = 0.130). UCA alone did not outperform the Bishop score in predicting successful IOL. However, UCA combined with a Bishop score > 4 showed higher predictive performance for IOL success.	7/9
3	Xing et al., 2019 [14]	China	Retrospective cohort study	406	Development of a modified predictive scoring system to improve the safety and success rate of vaginal birth after cesarean section (VBAC).	N/A	Multivariable logistic regression identified several independent predictors of successful vaginal birth after cesarean delivery (VBAC), including a history of previous vaginal delivery (OR 2.5, 95% CI 1.8–3.8), maternal age under 40 years (OR 2.1, 95% CI 1.2–3.3), pregnancy weight gain of less than 20 kg (OR 1.5, 95% CI 1.2–2.3), absence of labor induction (OR 1.9, 95% CI 1.5–2.9), a high pelvis-to-birth weight score (OR 1.4, 95% CI 1.1–2.1), and a higher Bishop score (OR 1.3, 95% CI 1.2–1.4)	8/9
4	Wei et al., 2019 [15]	China	Prospective cohort study	1,408 (365 received oxytocin, 1,043 received vaginal dinoprostone)	Comparison of dinoprostone and oxytocin for induction of labor (IOL) at term, and assessment of cesarean section rates across ten centers in Southern China.	Oxytocin and vaginal dinoprostone	Among women with a Bishop score of 0–3, induction of labor with dinoprostone resulted in a significantly lower cesarean section rate compared to oxytocin (33.3% vs. 58.5%, p = 0.01), indicating superior efficacy of dinoprostone in this subgroup. In contrast, among women with a Bishop score of 4–6, the mode of induction—whether dinoprostone or oxytocin—did not significantly influence cesarean section rates (32.1% vs. 38.2%, p = 0.48). These findings suggest that dinoprostone may be the preferred agent for women with an unfavorable cervix (Bishop score ≤3), while either agent may be appropriate when the Bishop score is 4–6, particularly in settings where dinoprostone is not available.	8/9
5	Vallikkannu et al., 2017 [16]	Malaysia	Prospective cohort study	193	Evaluation of IGFBP-1 as a predictor of successful IOL, compared with the Bishop score and cervical length measured by transvaginal ultrasound (TVUS).	Dinoprostone for cervical ripening. Once the cervix was adequately dilated, amniotomy was performed followed by oxytocin infusion.	In nulliparous women undergoing induction of labor, cervicovaginal IGFBP-1 testing predicted vaginal delivery more accurately than either the Bishop score or transvaginal ultrasound (TVUS) cervical length. The sensitivity and specificity of IGFBP-1 were 82.2% and 87.5%, respectively, compared to 64.4% and 62.5% for the Bishop score, and 75.6% and 68.7% for TVUS. These findings suggest that IGFBP-1 testing may be a valuable adjunct in guiding decision-making for induction of labor in nulliparous women.	8/9
6	Uygur et al., 2016 [17]	Turkey	Prospective cohort study	73	Comparison of fetal fibronectin (fFN), ultrasound parameters, and the Bishop score in predicting successful IOL when the cervix is unfavorable.	Intravenous oxytocin started at 2 mU/min and increased by 2 mU/min every 15 minutes, with a maximum of 20 mU/min.	In women with an unfavorable cervix, defined as a Bishop score below 5, the presence of fetal fibronectin (fFN) in vaginal secretions was found to be a strong predictor of successful induction of labor. The presence of fFN was significantly associated with achieving vaginal delivery within 24 hours of induction. In contrast, neither cervical length assessed by transvaginal ultrasound nor the Bishop score demonstrated predictive value in this context. Logistic regression analysis confirmed that only fFN emerged as an independent and statistically significant predictor of successful labor induction, highlighting its potential clinical utility in guiding decision-making when considering induction in nulliparous women with an unripe cervix.	7/9
7	Taha et al., 2020 [18]	Egypt	Prospective cohort study	162	To evaluate the role of cervical length measurement in predicting successful vaginal delivery and its relationship with labor duration.	Vaginal prostaglandin E2	Antenatal cervical length measurement was significantly associated with both the mode of delivery and the gestational age at delivery. Women with shorter cervical lengths were more likely to achieve successful vaginal delivery (multivariate OR 0.89, 95% CI 0.84–0.95, p < 0.001). Additionally, cervical length was positively correlated with gestational age at delivery (ρ = 0.30, p = 0.01 in nulliparous women). These findings suggest that cervical length assessment may serve as a non-invasive and objective alternative to the Bishop score in counseling patients about the likelihood of vaginal delivery.	8/9
8	Silva et al., 2017 [19]	Brazil	Retrospective cohort study	412	To evaluate cases of labor induction with 25 μg vaginal misoprostol tablets and maternal outcomes at a tertiary hospital in southeastern Brazil.	Vaginal misoprostol	In a cohort of women undergoing induction of labor with vaginal misoprostol, only Bishop scores of 4 or 5 and a history of previous vaginal delivery emerged as independent predictors of successful vaginal delivery. Stepwise regression analysis confirmed that both factors were statistically significant, with β = 0.23 (p < 0.001) for Bishop scores of 4–5 and β = 0.22 (p < 0.001) for previous vaginal birth	7/9
9	Sievert et al., 2017 [20]	USA	Retrospective cohort study	331	To identify predictors of vaginal delivery in medically indicated early labor induction.	Labor typically initiated with Foley catheter and simultaneous oxytocin infusion.	In women undergoing medically indicated early preterm induction of labor before 34 weeks of gestation, significant independent predictors of cesarean delivery included earlier gestational age (OR 0.79, 95% CI 0.71–0.88), a simplified Bishop score <4 (OR 0.58, 95% CI 0.42–0.79), suspected intrauterine growth restriction (OR 1.87, 95% CI 0.77–4.54), chronic hypertension (OR 2.53, 95% CI 1.40–4.58), and higher maternal body mass index (OR 1.03, 95% CI 1.00–1.07.	7/9
10	Sevrin et al., 2019 [21]	Brazil	Prospective cohort study	80	To evaluate the use of transvaginal ultrasound (TVUS) of the cervix as a tool for predicting labor outcomes in prolonged pregnancy.	For labor induction (Bishop score < 6), 5 IU oxytocin was administered starting at 2 mIU/min (4 drops/min) with 2 mIU increases every 30 minutes until effective contractions began. Maximum dose was 32 mIU/min (64 drops/min). Artificial rupture of membranes was also used as an alternative.	Among the variables and combinations analyzed, a cervical length of 3 cm and a Bishop score ≤2 were identified as the strongest independent predictors of failed induction of labor. These parameters demonstrated the highest discriminative capacity in predicting induction failure, supporting their utility in pre-induction assessment and clinical decision-making.	9/9
11	Sahin et al., 2022 [22]	Turkey	Retrospective cohort study	474	To present a case series of labor induction after cesarean section (TOLAC) and identify significant predictors of VBAC success.	For women with more than two contractions during a 10-minute cardiotocographic observation, balloon catheter was preferred over oxytocin. Oxytocin was indicated for fewer than three contractions in 10 minutes. Five units of oxytocin in 1 liter of saline were administered.	Significant predictors of successful vaginal birth after cesarean (VBAC) included the Bishop score at admission, body mass index (BMI), number of previous vaginal deliveries, and estimated fetal weight. Women with a BMI < 29 kg/m² had nearly double the likelihood of successful VBAC compared to those with higher BMI (OR 1.92, 95% CI 1.33–2.78, p < 0.001). Prior vaginal delivery was associated with a reduced risk of failed trial of labor (RR 0.4, 95% CI 0.2–0.6, p < 0.001), and fetal weight < 4,000 g significantly increased the probability of vaginal delivery. The Bishop score demonstrated the strongest predictive value (AUC = 0.799, p < 0.001), followed by the TOLAC score (AUC = 0.666, p < 0.001)	8/9
12	Levin et al., 2022 [23]	Israel	Retrospective cohort study	413	To determine factors associated with successful IOL after cesarean section in multiparous women.	Oxytocin	Only the duration of membrane rupture and the modified Bishop score were associated with successful IOL.	8/9
13	Bajpai et al., 2015 [24]	India	Prospective cohort study	131	To evaluate the role of transvaginal sonographic (TVS) assessment of the cervix before induction in predicting delivery outcome and compare its performance to the Bishop score in IOL patients.	Intracervical application of 0.5 mg PGE2 gel. If no uterine contractions or cervical changes occurred within 8 hours, induction was repeated, up to three times in 24 hours. If the Bishop score became favorable, amniotomy followed by 2 units of IV oxytocin at 2 mIU/min (8 drops/min) was given, gradually increased to 16 mIU/min (60 drops/min).	A 5-feature TVS-based score predicted induction success better than the Bishop score. Two features—longer cervical length and greater distance between presenting fetal part and external cervical os—were independently predictive of induction failure.	9/9
14	Oros et al., 2017 [25]	Spain	Prospective cohort study	245	To predict perinatal outcomes and healthcare costs following IOL in term pregnancies.	N/A	Lower Bishop scores prior to induction of labor and a history of previous vaginal deliveries were independently associated with increased total hospital costs. Specifically, patients with a Bishop score <5 incurred significantly higher induction-related expenses, and multiparous women, despite shorter labor durations, had higher overall charges—primarily driven by increased use of inpatient resources and interventions (p < 0.001 for both variables)	8/9
15	Obeidat et al., 2021 [26]	Jordan	Prospective cohort study	330	To evaluate predictors of vaginal delivery in both nulliparous and multiparous women in Northern Jordan after IOL with vaginal prostaglandins.	Vaginal prostaglandins	In nulliparous women, higher Bishop scores at admission were significantly associated with increased rates of vaginal delivery (p < 0.001). Among multiparous women, vaginal delivery rates also rose with increasing Bishop scores (p = 0.001), while a higher body mass index (BMI) was independently associated with a reduced likelihood of vaginal delivery (p = 0.003)	7/9
16	Navve et al., 2017 [27]	Israel	Retrospective cohort study	600	To determine whether the Bishop score before induction can predict: (1) the mode of delivery, and (2) maternal and neonatal outcomes in multiparous women.	Oxytocin, a slow-release vaginal PGE2 insert (10 mg dinoprostone), or a double-balloon transcervical catheter.	In multiparous women undergoing induction of labor after 34 weeks of gestation, the initial Bishop score did not significantly influence the likelihood of successful vaginal delivery or the incidence of maternal and neonatal complications. The vaginal delivery rate exceeded 93% in both the low Bishop score group (<6) and the high score group (≥6), with no statistically significant differences in cesarean delivery rates (3.7% vs. 3.2%, p = NS), vacuum extraction, or adverse maternal and neonatal outcomes. These findings indicate that induction of labor in multiparous women is both safe and highly successful, irrespective of cervical favorability as assessed by the Bishop score.	8/9
17	Bila et al., 2020 [28]	Serbia	Prospective cohort study	146	To evaluate the simplified Bishop score and ultrasound cervical measurement in predicting successful IOL in nulliparous women.	IV oxytocin, prostaglandin E1 tablets, or prostaglandin E2 tablets.	The study demonstrated that a simplified Bishop score greater than 5 and a shorter cervical length measured by transvaginal ultrasound were both significant predictors of successful induction of labor in nulliparous women. Specifically, a simplified Bishop score >5 had a sensitivity of 81.4% and specificity of 73.3%, while cervical length ≤25 mm had a sensitivity of 79.1% and specificity of 76.6%, confirming the utility of both measures in predicting favorable outcomes following IOL in this population	9/9
18	Brik et al., 2017 [29]	Spain	Prospective cohort study	179	To identify predictors of failed induction of labor during pregnancy.	Dinoprostone and oxytocin, or dinoprostone alone.	Cervical length measured before induction of labor was found to be an independent and significant predictor of induction failure, with a length ≥28 mm associated with a markedly higher risk of failed induction compared to shorter lengths. When combined with a Bishop score ≤3, the predictive value for failure increased substantially, suggesting that cervical length assessment may offer a valuable, objective adjunct to Bishop scoring for evaluating the likelihood of successful induction in term pregnancies	9/9
19	Bashirudin et al., 2023 [30]	Malaysia	Retrospective cohort study	819	To identify independent predictors of successful IOL in pregnant women with a history of cesarean section.	Balloon catheter, vaginal dinoprostone, or oxytocin.	BMI, height, ethnicity, number of deliveries, reason for previous cesarean section, and the Bishop score were independent predictors of IOL success.	8/9
20	Khandelwal et al., 2018 [31]	India	Prospective cohort study	66	To compare the Bishop score and cervical length via transvaginal ultrasound (TVUS) for predicting active labor within 6 hours, time from induction to delivery, and duration of active labor, and to identify optimal cut-off points for each method.	Foley catheter insertion under direct vision. Vaginal misoprostol 25 mcg every 4 hours. Labor management followed hospital protocol, with amniotomy and/or IV oxytocin if contractions were inadequate. Oxytocin started 6 hours after last misoprostol dose and titrated up every 20 minutes.	The optimal Bishop score cut-off for predicting IOL response within 6 hours was > 4 (sensitivity 69%, specificity 79%). For cervical length, ≤ 25 mm was the best cut-off (sensitivity 51%, specificity 70%). The Bishop score outperformed cervical length measured by TVUS in predicting labor induction response.	9/9
21	Facchinetti et al., 2015 [32]	Italy	Prospective cohort study	234	To evaluate factors predicting the likelihood of successful IOL in women with a previous cesarean section.	Artificial rupture of membranes, oxytocin infusion, dinoprostone, or a combination of these.	In women undergoing induction of labor for premature rupture of membranes (PROM), multivariate analysis identified history of previous vaginal delivery (OR 3.0, 95% CI 1.5–5.8), non-African ethnicity (OR 2.3, 95% CI 1.2–4.3), and a Bishop score ≥5 at admission (OR 2.6, 95% CI 1.4–5.1) as significant independent predictors of successful vaginal delivery.	9/9
22	De Miguel Manso et al., 2020 [33]	Spain	Prospective cohort study	231	Validation of a pilot prediction model performed by a single observer, based on clinical and ultrasound parameters.	Dinoprostone or oxytocin	In the external validation cohort, only ultrasound parameters—fetal head–perineum distance (FHPD) and cervical length (CL)—and the Bishop score were significantly associated with the outcome of labor induction. Women who achieved vaginal delivery had a significantly shorter median FHPD (41 mm [IQR 35–48]) compared to those who underwent cesarean section (53 mm [IQR 48–58], p < 0.001). Similarly, cervical length was shorter in the vaginal delivery group (27 mm [IQR 22–32]) than in the cesarean group (29 mm [IQR 26–35], p = 0.04). Bishop scores were also significantly higher among women who delivered vaginally (median 5 vs. 3, p < 0.001). These three parameters—FHPD, CL, and Bishop score—were the only significant predictors of induction outcome in multivariate analysis.	8/9
23	Lin et al., 2019 [34]	China	Prospective cohort study	162	To investigate factors that can be used to predict successful vaginal delivery after cesarean section and related outcomes.	Oxytocin, artificial rupture of membranes, and mechanical cervical dilation.	Both the Bishop score at admission and the presence of spontaneous labor were independently associated with successful vaginal birth after cesarean section (VBAC). Multivariate logistic regression revealed that a Bishop score ≥6 significantly increased the likelihood of vaginal delivery (adjusted OR 4.00, 95% CI 2.20–7.28, p < 0.001). Additionally, the occurrence of spontaneous labor was an independent positive predictor of VBAC success (adjusted OR 2.21, 95% CI 1.16–4.21, p = 0.016).	8/9
24	Ivars et al., 2016 [35]	France	Retrospective cohort study	326	To confirm the predictive value of parity for successful induction and propose improvements to the Bishop score by including parity and simplifying the original score.	Combination of oxytocin (in the absence of active labor) and amniotomy (if membranes were intact).	In women undergoing induction of labor with oxytocin and amniotomy, cervical position and consistency were not significant predictors of successful vaginal delivery. Instead, the study confirmed the utility of a simplified Bishop score that excluded those parameters and incorporated parity. This modified score demonstrated strong predictive performance, with a vaginal delivery rate of 91% in women scoring ≥5, compared to 67% in those scoring <5 (p < 0.001). Parity was the strongest individual predictor, with multiparous women significantly more likely to deliver vaginally than nulliparous women (OR 3.6, 95% CI 2.4–5.3, p < 0.001).	9/9
25	Ma et al., 2023 [36]	China	Prospective cohort study	233	To evaluate predictors of vaginal delivery after balloon catheter IOL in women with a prior cesarean section and an unfavorable cervix.	Cervical ripening using a balloon catheter.	Lower estimated fetal weight was independently associated with a higher likelihood of vaginal birth after cesarean section (VBAC). Multivariate logistic regression analysis showed that for each 100 g decrease in fetal weight, the probability of successful VBAC increased significantly (adjusted OR 1.13, 95% CI 1.06–1.20, p < 0.001), highlighting fetal weight as a key predictor in the decision-making process for trial of labor after cesarean.	9/9
26	Gokturk et al., 2015 [37]	Turkey	Prospective cohort study	223	To evaluate cervical length, posterior cervical angle, and fetal head position via ultrasound as alternative predictors of successful IOL compared to the Bishop score.	Oxytocin infusion. Amniotomy was performed in patients who had regular contractions with cervical dilation < 4 cm.	. In women undergoing induction of labor, multiparity, shorter cervical length, wider posterior cervical angle (PCA), and higher Bishop scores were all significant predictors of successful vaginal delivery. Multivariate logistic regression identified Bishop score (OR 2.38, 95% CI 1.38–4.11, p = 0.002), cervical length (OR 0.88, 95% CI 0.81–0.95, p = 0.001), PCA (OR 1.06, 95% CI 1.01–1.11, p = 0.012), and parity (OR 4.14, 95% CI 1.52–11.29, p = 0.005) as independent predictors of successful induction. In contrast, fetal head position showed no significant association with delivery outcome (p = 0.79).	9/9
27	Abdullah et al., 2022 [38]	Malaysia	Observational comparative study	294	To assess the association between transvaginal ultrasound (TVUS) cervical assessment and Bishop score for predicting successful IOL, including optimal cut-off points and patient acceptance of both methods.	Intracervical balloon catheter, prostaglandin E2 tablets, IV oxytocin, or combinations of these methods.	TVUS assessment of cervical length demonstrated predictive accuracy comparable to that of the Bishop score in forecasting successful induction of labor. Receiver operating characteristic (ROC) curve analysis showed an AUC of 0.701 for cervical length and 0.719 for the Bishop score, indicating similar discriminative ability. The optimal cut-off values identified were >27 mm for cervical length (sensitivity 76.7%, specificity 64.4%) and >4 for the Bishop score (sensitivity 86.7%, specificity 60%)	9/9
28	Marciniak et al., 2020 [39]	Poland	Retrospective cohort study	327	To identify predictors of cesarean delivery in patients with an unfavorable cervix undergoing cervical ripening and IOL using a Foley catheter.	Foley catheter. Upon removal, IV oxytocin was initiated at 6 mU/min, increased every 20–25 minutes, up to a maximum of 25 mU/min or until satisfactory contractions occurred.	Among term pregnant women undergoing induction of labor, a Bishop score of 1–2 was independently associated with an increased risk of cesarean delivery (OR 2.28, 95% CI 1.34–3.88), while a Bishop score of 3–4 acted as a protective factor, significantly reducing the likelihood of cesarean delivery (OR 0.44, 95% CI 0.25–0.76) compared to women with a score of 0.	8/9
29	Al-Adwy et al., 2018 [40]	Egypt	Prospective observational study	70	To determine the accuracy of the posterior cervical angle (PCA) compared with cervical length and Bishop score in predicting IOL outcome.	Initial cervical ripening with 25 μg vaginal misoprostol. Cervix reassessed after 6 hours to decide on repeat dosing or IV oxytocin.	In predicting successful induction of labor, the optimal cut-off points identified were a posterior cervical angle (PCA) > 99.5° (sensitivity 91.8%, specificity 90.5%, AUC 0.94), cervical length 5 (sensitivity 73.5%, specificity 81.0%, AUC 0.82). Among these, the PCA demonstrated the highest predictive accuracy with a positive likelihood ratio of 9.64 and negative likelihood ratio of 0.09.	9/9
30	Lueth et al., 2020 [41]	Ethiopia	Prospective cohort study	346	To assess the prevalence, outcomes, and associated factors of labor induction.	For unfavorable cervix (Bishop score < 4), 25–50 μg vaginal misoprostol was administered every 6 hours up to a maximum of 200 μg. For severe oligohydramnios, a balloon catheter was used.	The post-cervical ripening Bishop score remained a significant independent predictor of successful induction of labor. Women who achieved a favorable Bishop score (≥4) after ripening were 8.28 times more likely to experience a successful vaginal delivery compared to those with scores <4 (adjusted OR 8.28, 95% CI 4.31–15.90, p < 0.001).	8/9
31	Kawakita et al., 2020 [42]	USA	Retrospective cohort study	835	To validate a predictive model for calculating the probability of vaginal delivery after induction with an unfavorable cervix.	N/A	In women undergoing induction of labor at 39 weeks, factors independently associated with successful vaginal delivery included advanced gestational age at induction (adjusted OR 1.38 per additional week, 95% CI 1.22–1.56), multiparity (aOR 2.36, 95% CI 1.56–3.57), simplified Bishop score >4 (aOR 2.15, 95% CI 1.62–2.85), and the presence of premature rupture of membranes (aOR 1.65, 95% CI 1.18–2.31).	9/9
32	Alvarez-Colomo et al., 2016 [43]	Spain	Prospective observational study	151	To evaluate the validity of ultrasound in predicting IOL outcomes compared to the Bishop score, and to develop a prediction model incorporating ultrasound and clinical variables.	If the Bishop score > 7, IOL was initiated early with amniotomy and oxytocin infusion. If labor did not occur, the woman was reassessed after 24 hours and additional dinoprostone was administered if needed.	Fetal head–perineum distance (FHPD) and cervical length (CL) were found to be significant predictors of delivery mode, showing comparable predictive ability to the Bishop score. The area under the ROC curve was highest for FHPD (AUC 0.734), followed by the Bishop score (AUC 0.678) and cervical length (AUC 0.663). An FHPD > 45 mm and CL > 25 mm were associated with increased risk of cesarean delivery.	9/9
33	Lu et al., 2020 [44]	China	Prospective cohort study	475	To assess the predictive value of cervical shear wave elastography on IOL outcomes.	If Bishop score ≥ 6, the cervix was considered favorable and IOL proceeded with amniotomy and/or oxytocin; if < 6, vaginal prostaglandin E2 was used.	Multivariate analysis showed that lower elasticity values near the internal os (measured by shear wave elastography) and longer cervical length significantly increased the risk of cesarean delivery. Prediction models incorporating shear wave elastography achieved higher diagnostic accuracy than those based on the Bishop score alone, with an AUC of 0.867 for the elastography model versus 0.678 for the Bishop score (p < 0.001), confirming the superior predictive performance of quantitative elastographic assessment	7/9
34	Chan et al., 2019 [45]	China	Prospective cohort study	308	To examine (1) agreement between manual and automated measurement of parasyphysis angle of progress (psAOP), (2) reproducibility of various ultrasound parameters, and (3) the value of transperineal ultrasound (TPU) in predicting IOL outcomes.	IOL by amniotomy followed by oxytocin if Bishop score ≥ 6. If unfavorable, 10 mg vaginal dinoprostone was used. Intracervical balloon was used for prior cesarean. Reassessment at 24 hrs guided further treatment.	Multivariate logistic regression revealed that maternal age (OR 1.06, 95% CI 1.01–1.11, p = 0.017), history of prior cesarean section (OR 3.28, 95% CI 1.67–6.45, p = 0.001), a low Bishop score (OR 0.75, 95% CI 0.65–0.87, p < 0.001), and a low pressure-stretch adjusted opening point (psAOP) (OR 0.93, 95% CI 0.89–0.97, p = 0.001) were all significant independent predictors of cesarean delivery following induction of labor.	9/9
35	Krsman et al., 2023 [46]	Serbia	Prospective cohort study	192	To develop a machine learning-based model to predict IOL success.	If Bishop ≤ 5, IOL started with intracervical dinoprostone gel (0.5 mg/3 g, 2.5 ml gel). If Bishop score ≥ 6, IOL was initiated with IV oxytocin.	A machine learning model incorporating clinical variables (parity, BMI), transvaginal ultrasound parameters (such as cervical length and posterior cervical angle), and the Bishop score significantly outperformed traditional prediction models in forecasting successful induction of labor. The combined model achieved an area under the ROC curve (AUC) of 0.86, compared to 0.69 for the Bishop score alone and 0.74 for the model using only clinical and ultrasound variables, demonstrating the enhanced predictive power of integrated, machine learning–driven approaches.	6/9
36	Liu et al., 2023 [47]	China	Prospective cohort study	101	To assess whether combining ultrasound data and machine learning with the Bishop score improves cervical maturity assessment accuracy.	Oxytocin, or oxytocin with misoprostol, or misoprostol, oxytocin, and amniotomy.	Machine learning–based assessment of cervical maturity using transvaginal ultrasound parameters proved to be more objective and accurate than the traditional Bishop score in predicting cervical ripeness. The machine learning model demonstrated superior predictive performance, with an AUC of 0.88 compared to 0.69 for the Bishop score (p < 0.001). Additionally, the model showed higher sensitivity (84.3% vs. 63.4%) and specificity (83.6% vs. 68.4%), confirming its clinical utility as a more reliable tool for pre-induction evaluation.	7/9

Based on the detailed data presented in the referenced table, findings were extracted and organized to address key issues related to the Bishop score, cervical ripening, labor induction techniques, and the prognostic value of the Bishop score in predicting successful induction of labor.

Bishop Score and the Need for Cervical Ripening

Cervical ripening is widely recommended prior to labor induction when the Bishop score is less than 6, as it enhances the likelihood of vaginal delivery and reduces cesarean section risk. The Bishop score serves as a key prognostic tool for assessing cervical readiness and determining the necessity of ripening interventions.

Several studies support defined threshold values below which cervical ripening is particularly beneficial (Table 2).

**Table 2:** Data on Bishop Score and the Need for Cervical Ripening BMI: body mass index

Study (Ref)	Bishop Score Range	Finding	Implication
Zelig [12]	≤5	Score of 5 optimal for predicting successful induction in nulliparous women, regardless of BMI	Scores <5 suggest need for cervical preparation
Wei [15]	0–3	Dinoprostone more effective than oxytocin in low-score patients	Supports pharmacologic ripening when Bishop score is low
Sevrin [21]	≤2	Low Bishop score and cervical length <3 cm predicted failure	Useful criteria for ripening candidate selection
Marciniak [39]	≤3	Significantly associated with unsuccessful induction	Reinforces ripening necessity for low scores
Lueth [41]	<4 vs. ≥4 (post-ripening)	Score ≥4 after ripening linked to higher success	Post-ripening score ≥4 should be a target before induction

Collectively, these findings affirm the Bishop score as a central element in guiding labor induction strategies. When the score is <6, particularly at or below thresholds of 3-5, cervical ripening is strongly indicated to enhance the probability of a successful induction and minimize maternal and neonatal complications.

Effectiveness of Cervical Ripening Techniques According to Bishop Score

Comparative evaluations of cervical ripening strategies, encompassing pharmacological agents, mechanical devices, and novel predictive tools, highlight the importance of tailoring interventions based on initial Bishop scores. For women presenting with an unfavorable cervix (typically a Bishop score ≤3), evidence consistently supports the use of specific techniques to enhance induction success and reduce adverse outcomes (Table 3).

**Table 3:** Effectiveness of Cervical Ripening Techniques According to Bishop Score. CL: cervical length;  IGFBP1: insulin-like growth factor binding protein-1

Study (Ref)	Bishop Score Range	Technique	Finding	Implication
Wei et al. [15]	0–3	Dinoprostone vs. oxytocin	Dinoprostone superior in low-score scenarios	Prostaglandins preferred when Bishop score is very low
Silva et al. [19]	4–5	Vaginal misoprostol	Misoprostol effective; prior vaginal delivery improved outcomes	Both cervical score and obstetric history are important
Vallikkannu et al. [16]	<6	IGFBP-1, transvaginal ultrasound	IGFBP-1 predicted better than Bishop or CL	Emerging biomarkers may enhance prediction
Ma et al. [36]	Unfavorable	Balloon catheter	Effective in prior cesarean with low Bishop score	Mechanical methods useful in specific populations
Lueth et al. [41]	≥4 (post-ripening)	Misoprostol or balloon	Score ≥4 post-ripening associated with success	Post-ripening score is a key treatment threshold

These findings collectively affirm that the effectiveness of cervical ripening techniques is closely linked to baseline cervical status. Prostaglandins are particularly beneficial in low Bishop score settings, while mechanical approaches offer viable alternatives in select populations. Achieving a favorable post-ripening Bishop score remains a key determinant of induction success, guiding the choice and timing of subsequent interventions.

Induction Techniques Based on Bishop Score

The effectiveness of labor induction techniques is closely linked to the initial Bishop score, which serves as a critical parameter for selecting the most appropriate method. Tailoring induction strategies to cervical status not only improves the likelihood of vaginal delivery but also reduces the risk of unnecessary interventions, including cesarean section.

Evidence supports the stratification of induction methods by Bishop score thresholds (Table 4).

**Table 4:** Induction Techniques Based on the Bishop Score. IGFBP1: insulin-like growth factor binding protein-1; UCA: uterocervical angle

Study (Ref)	Bishop Score Range	Technique	Finding	Implication
Yang [13]	<4 vs. ≥4	Dinoprostone vs. Oxytocin + UCA	Dual-parameter (Bishop + UCA) improves induction planning	Stratified induction based on combined score and imaging
Vallikkannu [16]	<6	Sequential Dinoprostone, Amniotomy, Oxytocin + IGFBP-1	IGFBP-1 may outperform Bishop score alone	Support for multimodal prediction using biomarkers
Silva [19]	4–5	Misoprostol	Scores 4–5 linked to successful induction	Prostaglandins effective in borderline cervices
Lu [44]	≥6	Amniotomy and/or Oxytocin	Effective in favorable cervices without ripening	Less intervention needed with high Bishop score
Krsman [46]	≤5 vs. ≥6	Machine Learning Model	ML model integrating Bishop and ultrasound improved forecasting	Supports personalized medicine in induction planning

Altogether, these findings advocate for a stratified and multimodal approach to labor induction. Bishop score remains foundational, but optimal outcomes are achieved when it is complemented by additional clinical and imaging data to refine the choice of induction method.

Predictive Value of Bishop Score for Successful Labor Induction

Bishop score remains a cornerstone in predicting the success of labor induction, serving both as a standalone clinical tool and as a component of more complex predictive models. It informs key decisions about induction strategy, patient counseling, and expectations for delivery outcomes. Multiple studies affirm the Bishop score’s prognostic significance across diverse patient populations (Table 5).

**Table 5:** Predictive Value of the Bishop Score for Successful Labor Induction. BMI: body mass index; CL: cervical length; fFN: fetal fibronectin; ML: machine learning; PCA: posterior cervical angle; TVUS: transvaginal ultrasound; UCA: uterocervical angle; US: ultrasound

Study (Ref)	Bishop Score Range	Predictive Tool	Key Finding	Adjunct Predictors
Zelig [12]	≤5	Bishop score	Score of 5 optimal threshold in nulliparous women	BMI (no effect)
Yang [13]	Higher vs. lower	Bishop score + UCA	Higher scores + UCA = improved prediction	Uterocervical angle
Wei [15]	0–3	Bishop score	Dinoprostone is better for low scores	None
Silva [19]	4–5	Bishop score + history	Scores 4–5 and prior vaginal birth predicted success	Obstetric history
Uygur [17]	<5	Bishop score + fFN	fFN predicted success in low-score women	Fetal fibronectin (fFN)
Taha [18], Sevrin [21]	Low	Bishop score + CL	Low score + short CL predicted failure	Cervical length (TVUS)
Bila [28]	Modified	Simplified Bishop + US	Improved prediction in nulliparous women	Ultrasound
Brik [29], Kawakita [42]	≤3 vs. >4	Bishop score	Low score = failure; >4 = success in multiparas	Parity
Khandelwal [31]	All	Bishop score vs. CL	Bishop more reliable than CL	Cervical length
Gokturk [37]	All	Ultrasound parameters	CL, PCA, parity predictive—not fetal head position	CL, PCA, parity
Abdullah [38], Alvarez-Colomo [43]	All	CL, Head–perineum distance	US measurements comparable to Bishop score	Ultrasound metrics
Lu [44]	All	Shear-wave elastography	Stiffness assessment aligned with Bishop	Elastography
Chan [45], Liu [47]	All	ML + Bishop + US	Improved individualized prediction	ML, US

The Role of Bishop Score in Predicting Vaginal Birth After Cesarean (VBAC)

Bishop score plays a crucial role in predicting the likelihood of successful vaginal birth after cesarean section (VBAC), serving as a key parameter in individualized risk stratification and induction planning for women with a history of cesarean delivery. Its integration into predictive models enhances clinical decision-making and contributes to safer, more effective VBAC management (Table 6).

**Table 6:** Role of Bishop Score in Predicting VBAC. BMI: body mass index; CS: cesarean section; GWG: gestational weight gain; PROM: premature rupture of membranes; VBAC: vaginal birth after cesarean

Study (Ref)	Bishop Score Use	Key Finding	Other Predictors
Xing [14]	Included in prediction model	Favorable score predicted successful VBAC	Prior vaginal birth, maternal age <40, low GWG, no induction, pelvic-birthweight ratio
Bashirudin [30]	Independent predictor	Score, BMI, ethnicity, parity, prior CS indication all predicted VBAC success	BMI, ethnicity, parity, indication for prior CS
Facchinetti [32]	Score ≥5	Score ≥5, prior vaginal birth, non-African ancestry linked to VBAC success	History of vaginal delivery, ethnicity, PROM induction
Lin [34], Levin [12]	Combined with obstetric history	Score, BMI, prior vaginal births predicted VBAC success in multiparas	Prior vaginal births, BMI

These studies collectively underscore the importance of combining clinical history with cervical assessment to guide counseling and induction strategies in women pursuing VBAC.

Role of Bishop Score in Perinatal Outcomes and Health Care Costs

The implications of the Bishop score extend beyond clinical outcomes to include economic impacts and perinatal health indicators. Oros et al. [25] aimed to predict perinatal outcomes and healthcare costs in term pregnancies undergoing labor induction. Their study identified low Bishop scores and the absence of prior vaginal deliveries as factors significantly associated with increased induction-related healthcare costs. These findings underscore the potential economic burden of initiating labor induction in women with unfavorable cervical conditions and suggest that Bishop score-based strategies could contribute to more cost-effective obstetric care [25].

Discussion

This review provides a comprehensive synthesis of current evidence regarding the predictive value of the Bishop Score in assessing cervical readiness for labor induction, its impact on induction techniques, and its association with perinatal outcomes. Our findings affirm the enduring clinical relevance of the Bishop Score while also acknowledging the evolving methodologies and adjunctive tools that have emerged in the past decade.

Bishop Score remains a fundamental obstetric tool, widely used to evaluate cervical favorability prior to induction of labor. Consistent with prior research, the present review reinforces its predictive utility for successful vaginal delivery. A comprehensive meta-analysis by Teixeira et al. assessed 59 studies examining the association between Bishop Score and vaginal birth outcomes, demonstrating that each unit increase in the Bishop score significantly improved the odds of successful vaginal delivery, both in general and within specific time frames (summary odds ratio [sOR] = 1.33, 95% CI: 1.13-1.56; sOR = 1.52, 95% CI: 1.37-1.70) [48].

Similarly, a prior meta-analysis by Crane et al., which evaluated data up to 2005, found that both transvaginal ultrasound and fetal fibronectin were predictive of successful labor induction but were not superior to the Bishop Score [49]. These findings are echoed by more recent studies included in this review, such as those by Uygur et al. [17], Abdullah et al. [38], and Alvarez-Colomo et al. [43], who also concluded that while sonographic and biochemical markers show promise, they do not surpass Bishop Score in prognostic accuracy.

Kolkman et al. conducted a systematic review involving over 13,000 women to evaluate the predictive value of the Bishop Score for cesarean delivery outcomes following labor induction. Their findings revealed poor diagnostic accuracy, with sensitivity-specificity combinations suggesting low specificity for cesarean prediction, even at higher score thresholds [50]. These results cast doubt on Bishop Score’s utility in forecasting delivery routes and call for re-evaluation of its role in modern practice.

The divergence between the findings of Kolkman et al. [50] and Teixeira et al. [48] likely stems from methodological and analytical differences. Kolkman’s analysis focused on cesarean prediction across a heterogeneous cohort without standardization of induction protocols, whereas Teixeira’s approach encompassed a broader range of outcomes and utilized a random-effects meta-analytic model accounting for confounders. The inconsistency also highlights the influence of study heterogeneity and quality assessment in shaping pooled estimates.

Kuba et al. critically re-examined the relevance of Bishop Score in contemporary obstetric settings, emphasizing that the original scoring system was developed over 60 years ago in a multiparous population with different induction agents (e.g., oxytocin and amniotomy) [6]. In contrast, modern obstetrics frequently involves induction in nulliparous women, late preterm pregnancies, and populations with diverse characteristics for whom the original scoring system may no longer be optimal. Moreover, the biological processes of cervical remodeling may not be adequately captured by the Bishop Score, as suggested by recent data.

Evidence from the ARRIVE trial further challenges the notion that low Bishop Scores are invariably associated with failed inductions or cesarean delivery. Modern pharmacological agents and mechanical ripening methods can achieve successful vaginal birth even with lower initial scores, indicating that reliance on Bishop score alone may underestimate cervical potential [7, 51].

This review also identifies key limitations in the available literature, including publication bias favoring positive results, selection bias during study inclusion, and significant heterogeneity across populations, settings, and interventions. Differences in induction protocols, dosing, and outcome definitions further complicate comparative synthesis.

Despite these limitations, Bishop Score continues to serve as a valuable tool in induction decision-making. However, integrating it with emerging predictive markers, such as cervical ultrasound features, fetal fibronectin, shear-wave elastography, and machine learning models, may enhance its predictive power [45,46].

Future research should focus on the prospective validation of emerging predictive tools, including machine learning-based models and quantitative ultrasound parameters, in diverse obstetric populations. Larger multicenter studies are needed to assess the generalizability and clinical utility of these models across varying maternal characteristics, such as parity, BMI, ethnicity, and comorbidities. Additionally, the integration of objective, technology-driven metrics (e.g., cervical elastography, fetal head-perineum distance) into standardized induction protocols may improve consistency in clinical decision-making. While this study did not require advanced statistical modeling, incorporating complex modeling frameworks in future analyses, particularly for meta-analyses or subgroup comparisons, would strengthen the robustness of findings. Finally, cost-effectiveness analyses and real-world implementation studies should be undertaken to evaluate the feasibility and impact of incorporating these predictive tools into routine prenatal care [51-53].

## Conclusions

In conclusion, the collective analysis of 36 studies underscores the multifaceted role of the Bishop score in determining the appropriateness of labor induction and the need for cervical ripening. A Bishop score below 6 generally indicates the necessity for cervical preparation, with varying induction methods, such as oxytocin, misoprostol, and mechanical techniques, demonstrating differential effectiveness depending on the initial score. This review highlights a significant association between Bishop Score values and the success rates of specific induction strategies, suggesting that a tailored approach based on initial cervical assessment may optimize induction outcomes.

Moreover, the incorporation of additional predictive factors, such as the ultrasound-measured cervical angle and cervical length, alongside the traditional Bishop score, may enhance the accuracy of predicting successful induction. Advances in technology, particularly the integration of machine learning models that combine clinical and sonographic parameters with the Bishop score, show considerable promise in refining prediction and decision-making processes.

Importantly, patient tolerance and acceptance of various induction methods further emphasize the need for patient-centered care in clinical decision-making. This comprehensive analysis not only reaffirms the critical role of the Bishop score in guiding labor induction but also highlights the potential benefits of adopting multiparametric evaluation strategies, innovative biomarkers, and technological advancements to improve prediction and clinical outcomes.
